# 以紫癜性肾炎为首发表现的慢性淋巴细胞白血病1例

**DOI:** 10.3760/cma.j.cn121090-20260117-00032

**Published:** 2026-08

**Authors:** 梦维 仝, 卫霞 农, 大谦 章, 歆 潘

**Affiliations:** 石河子大学第一附属医院血液内科，石河子 832000 Department of Hematology, the First Affiliated Hospital of Shihezi University, Shihezi 832000, China

患者，男，70岁，以“诊断紫癜性肾炎2个月，发现白细胞升高2周”于2025年8月15日就诊我院血液科。2025年6月中下旬患者无诱因出现双下肢肿胀伴皮肤多发紫红色出血点，高于皮面，伴有瘙痒，尿中大量泡沫，就诊于石河子市人民医院，诊断为过敏性紫癜、紫癜性肾炎（具体依据不详），给予泼尼松60 mg每日1次、雷公藤1 mg·kg^−1^·d^−1^、呋塞米20 mg每日2次、螺内酯20 mg每日2次。后续患者于我院肾病科门诊规律随诊，激素逐渐减量，双下肢持续重度水肿及散在紫癜、大量蛋白尿、外周血白细胞计数进行性升高且见可疑细胞（7月29日WBC 38.0×10^9^/L、未见可疑细胞，8月15日WBC 55.7×10^9^/L、可疑细胞10％），肾病科医师建议血液科就诊。既往高血压病史15年。无家族史。

入院查体：皮肤巩膜无黄染，四肢、躯干皮肤布满点状褐色色素沉着，浅表淋巴结未触及肿大，扁桃体无肿大，心、肺、腹查体均未见异常，双下肢散在瘀点、瘀斑，双下肢重度水肿。辅助检查：全血细胞分类计数：WBC 55.7×10^9^/L，淋巴细胞占47％，单核细胞占3％，嗜酸性粒细胞占1％，嗜碱性粒细胞占1％，中性分叶核粒细胞占40％，中性杆状核粒细胞占2％，中性晚幼粒细胞占1％，中性中幼粒细胞占1％，可疑细胞占3％，反应性淋巴细胞占1％。24 h蛋白定量：6.21 g。β_2_微球蛋白3.2 mg/L。血清轻链：轻链κ 4.51 g/L，轻链λ 2.74 g/L，κ/λ 1.65。尿轻链：轻链λ 87.1 mg/L，轻链κ 11.60 mg/L。血清蛋白电泳：白蛋白53.2％，α_1_球蛋白5.9％，α_2_球蛋白18.7％，β_1_球蛋白5.7％，β_2_球蛋白10.4％，γ球蛋白6.1％，免疫固定电泳：未见单克隆条带（M蛋白阴性）。

骨髓形态学：增生活跃，淋巴细胞比例增高,占32％。流式细胞术免疫分析：存在约25.01％的异常单克隆B细胞群体，免疫表型：表达CD19、CD5，部分表达CD23、CD27，弱表达CD22，少量表达CD200，不表达CD81、CD79b、FMC7、CD20、CD10、CD38、CD34；表面免疫球蛋白轻链Kappa/Lambda表达不明显。RMH-CLL积分为5分，符合慢性淋巴细胞白血病/小淋巴细胞淋巴瘤（CLL/SLL）典型免疫表型。基因突变检测：TP53基因突变未见异常。骨髓未检测到IGHV区超突变（突变程度≤2％）。

腹膜后淋巴结超声检查：胰周、脐周、腹主动脉走行区及双侧髂血管周围未见明确肿大淋巴结。浅表淋巴结超声检查：颈部双侧、双侧腋窝区、双侧腹股沟区多发淋巴结显示。腹部常规超声检查（肝胆胰脾肾）：①肝囊肿；②胆囊壁毛糙；③双肾多发囊肿（左侧伴囊壁钙化）。

诊断：慢性淋巴细胞白血病（Binet分期：A期；Rai分期：Rai 0期；CLL-IPI评分：3分）。

予泽布替尼160 mg每日2次口服。第3个月随访：双下肢紫癜完全消退，无新发皮疹；双下肢水肿较前明显减轻，仅双侧足背部轻度凹陷性水肿（+）。无发热、关节痛、腹痛等不适。实验室检查：全血细胞分类计数：WBC 10.7×10^9^/L，中性粒细胞占62.2％，淋巴细胞占31.5％，单核细胞占5.2％，嗜酸性粒细胞占0.6％，嗜碱性粒细胞占0.5％。肝功能：总蛋白61.2 g/L，白蛋白33.3 g/L，球蛋白27.9 g/L。肾功能：肌酐118.7 µmol/L，尿素7.64 mmol/L，估算肾小球滤过率53.16 ml·min^−1^·（1.73 m^2^）^−1^。尿常规：尿蛋白（+++），潜血（+++），镜检：红细胞3～5/高倍镜视野（HP），白细胞2～3/HP。24 h蛋白定量3.77 g。第4个月随访：患者拒绝来院复查相关指标，电话随访自述一般情况良好，足背水肿已完全消退，无新发皮疹。因未能获得实验室数据，无法评估第4个月时CLL控制及肾损害改善情况。

讨论：CLL/SLL较少累及肾脏，相关文献多为个案报道，尤其以肾脏损害为首发表现者更为少见。综合本例临床特点，肾损害与CLL相关的可能性较大。其机制可能涉及单克隆免疫球蛋白介导的免疫复合物沉积或补体激活途径。

